# Global and Medial Temporal MRI Morphometry in Alzheimer’s Disease and Cognitively Normal Adults: A Retrospective Association Study

**DOI:** 10.3390/diagnostics16182975

**Published:** 2026-09-14

**Authors:** Charles Thompson, Hamsha Varsha, Tarun Goswami, for the Alzheimer’s Disease Neuroimaging Initiative

**Affiliations:** 1 Boonshoft School of Medicine, Wright State University, Dayton, OH 45435, USA; thompson.853@wright.edu; 2 Biomedical, Industrial, and Human Factors Engineering, Wright State University, Dayton, OH 45435, USA; somangalinramanathan.2@wright.edu

**Keywords:** ADNI, Alzheimer’s disease, magnetic resonance imaging (MRI), gray matter, white matter, neurodegeneration, medial temporal lobe

## Abstract

**Background/Objectives**: Alzheimer’s disease is one of the most prevalent forms of dementia and is accompanied by progressive anatomical changes within the brain. However, large-scale analyses that compare global and local anatomical differences across age, sex, diagnosis, and repeated scans remain limited. **Methods**: We constructed a cohort of 1385 total participants, with 3593 scans, who were diagnosed as cognitively normal or with Alzheimer’s at entry into the study. Global and medial temporal brain volumes from 3T MRI scans were normalized to intracranial volume, and we used a linear mixed-effects model to analyze the association between participants’ age, sex, and diagnosis while also accounting for multiple follow-up scans. Diagnosis-dependent relationships between global and medial temporal structures, brain parenchymal and hippocampal fractions, and body mass index were also calculated. **Results**: Older age was associated with a decrease in normalized brain volume across each region of interest. In general, AD participants had significantly lower volumes than cognitively normal individuals, with a greater degree of difference in medial temporal regions. Global measures did have different degrees of separation, with gray matter showing greater separation than white matter. Exponential model equations showed that the AD group generally had a greater age-associated percentage decrease across-ROI/ICV association. BMI nor BMI x diagnosis was significantly associated with brain volumes within the model, while the exploratory pooled pTau217 analysis showed it was 4.6 times higher in AD and negatively associated with most morphometric areas. **Conclusions**: This large, integrated analysis supports the use of MRI morphometry as a valuable tool to directly compare AD-associated differences across global and medial temporal areas and also evaluate diagnosis-dependent relationships across anatomically related structures. Findings indicate that medial temporal measures provide a greater AD vs. CN separation than global measures, with GM being more sensitive than WM. Plasma pTau217 also gives biological context, which helps suggest that MRI results should be interpreted with multiple clinical and biological factors.

## 1. Introduction

Alzheimer’s disease (AD) is a progressive neurodegenerative form of dementia and is often associated with cognitive decline, memory issues, and structural alterations in brain morphology [1]. AD itself accounts for up to 60–70% of dementia cases, which has been recognized as a public health issue because of the growing number of cases due to processes such as population aging [2,3]. The number of cases of dementia is projected to increase to 82 million in 2030 and just over 150 million in 2050 [4,5]. Therefore, utilizing evidence-based imaging techniques, such as magnetic resonance imaging (MRI), and other biological markers as biomarkers will prove significant in diagnosing, monitoring, and tracking disease progression.

MRI is a widely accessible and clinically useful imaging tool that can provide quantitative volumetric insight into a patient’s brain in vivo. This relatively noninvasive process allows both clinicians and researchers to examine atrophy-related changes over time, making MRI a stronger disease-course biomarker rather than a neuropathological biomarker.

In addition to neuroimaging measures, several biological biomarkers have been developed to improve the detection and characterization of Alzheimer’s disease [6]. In particular, the use of blood-based biomarkers has been increasing in prominence as a more practical alternative to CSF, being more readily available, accessible, inexpensive, and less invasive. Therefore, such testing may provide an inexpensive yet reliable means for longitudinal monitoring throughout the evolution of the disease [7]. Of these, plasma phosphorylated tau 217 (pT217) is a promising blood-based marker, showing significant relationships with tau PET, amyloid burden, and disease severity in several cohorts [7,8]. For the present study, the pT217 levels offer an additional marker of the underlying biological disease burden of AD. Therefore, the present study provides the opportunity to assess whether pT217 and MRI morphometric measures agree in their assessment of neurodegeneration in sites of tau accumulation.

Despite MRI morphometry being less biologically specific than amyloid-beta (Aβ), tau, cerebrospinal fluid (CSF), or plasma biomarkers, there is still great potential for measuring structural outcomes of AD-related pathology [9,10]. Therefore, MRI may offer additional information for clinicians to use to track and monitor disease progression or treatment viability, but it should be used in combination with pathological biomarkers within a diagnostic setting.

Most cases of AD are considered sporadic, with incidence doubling every 5-year age increase after the age of 65 [11]. Age and sex are thought to be important risk factors for AD [12]. Aging has been associated with volume loss, particularly in the medial temporal areas, and global gray matter (GM) and white matter (WM) loss, along with other three-dimensional changes in the brain [13,14,15,16]. Differences in sex may also further influence age-related structural integrity and the clinical presentation and risk for AD. Females tend to have a higher incidence of AD, and when diagnosed, have been reported to progress more quickly [17,18]. Additionally, about two-thirds of the people in the U.S. with AD are women, with women tending to live longer, supporting a higher prevalence of AD in women than in men [19,20,21]. Therefore, global and regional analyses of AD anatomical changes between biological males and females can offer more insight into whether age- and sex-related differences are uniform across the cohorts. Hormonal and genetic factors have been suggested in the general literature, but were not measured in the present study; the present study looks at sex as a demographic and risk-factor variable without the hormonal or genetic examination.

At this time, there are no known cures for AD, which is why understanding the disease’s temporal trends and characterizing its progression and anatomical differences is of importance. The present study uses MRI morphometric data of 1385 participants who underwent 3593 imaging scans to examine brain volumes associated with AD and cognitively normal (CN) individuals, with a specific focus on age, sex, diagnosis, and regional morphometric variables. By normalizing global and region-specific MRI data, we reduced confounds from differences in individual head size. Rather than evaluating each volume as a single measure, we performed a mixed-effects framework across multiple morphometric measures while accounting for repeated scans. We also modeled the diagnosis-dependent relationship between anatomically linked variables and examined brain parenchymal and hippocampal fractions to see if medial temporal loss was disproportionate to broader tissue loss. Other work has looked into how localized and global degenerative patterns present in different diagnostic groups, typically examining measures individually. The current study extends this by examining the proportionality of relative morphological changes in large-scale AD and CN groups. Using both linear mixed-effects models and global and regional direct comparisons, we statistically modeled the relative neurodegenerative volume differences among diagnostic groups and aimed to provide additional insight into MRI morphometry as an anatomical measure of disease burden. This provides an integrated approach for AD characterization within the realm of MRI biomarkers.

## 2. Methods

This retrospective observational study used data from the Alzheimer’s Disease Neuroimaging Initiative database, which was launched in 2003 under the leadership of Principal Investigator Michael W. Weiner, MD. One of the current goals of the project is to support the development of biomarkers for clinical trials.

### 2.1. Participant Selection

Participants (PTID) were selected and filtered using the Analysis Ready Cohort (ARC) Builder. Cohort selection was filtered based on the availability of required variables regarding the participants’ sex, diagnostic entry group, date of birth (DOB), 3T MRI scans, MRI exam date, and morphometric variables supplied by the UCSF–Cross-Sectional FreeSurfer 7.x (UCSFFSX7) data table. This data used the MRI processing software FreeSurfer, from either version 7.2.0 or 7.4.1, which both produce compatible results, as noted by the ARC Builder.

Participants were included if they were diagnosed as either AD or CN at entry into ADNI. Participants’ sex refers to sex assigned at birth as recorded in the participants’ original birth certificate, as referred to by PTGENDER within the dataset. Their diagnostic group was determined based on whether they were diagnosed as AD or CN when they entered the study. Entry diagnosis was used rather than diagnosis at each visit because conversion status (e.g., CN to AD) was not consistently available across all follow-up visits in the filtered cohort. Participants labeled as mildly cognitively impaired (MCI) were excluded as we did not want to assume they would develop AD with certainty. The goal with this methodology was to compare the morphological differences between clinically classified CN and AD participants.

After cohort selection, the total number of initial participants came out to be 1385, with an associated number of 3593 total initial scans. These numbers were determined after the selection criteria sorted the participants for the entire cohort. All available scans were kept for analysis to have a more robust dataset. Some scans did not contain all morphometric values for different regions of interest (ROI) and therefore scan sample sizes were slightly different for each morphometric parameter. Final participants and scans were reported separately for gray matter, white matter, hippocampus, and entorhinal cortex.

Once the cohort was filtered using the web-based ARC Builder available through the ADNA Image and Data Archive, the data was downloaded in Excel (version 16.112.4; Microsoft Corporation, Redmond, WA, USA).Individual participant data were then matched to their participant ID (PTID) for each of their corresponding MRI visits and ROI. Additionally, data on each participant’s date of birth (DOB) included only their birth month and year and not the specific day for privacy reasons. Each participant’s approximate age at the time of their MRI scan was calculated in Excel using the general formula seen in Equation (1). Thus, age at the time of the MRI scan was treated as a continuous variable:

(1) $$Age_{approximate}\;=\;\frac{MRI\;Exam\;Date\;-\;DOB}{365}$$

### 2.2. Morphometric Variables

The major morphometric variables collected from the UCSFFSX7 table, referred to as ROIs, were total gray matter (GM) volume, cerebral white matter (WM) volume, hippocampal volume, entorhinal cortex volume, and intracranial volume (ICV). Total GM and cerebral WM were chosen to reflect global parameters of volume loss, whereas the hippocampus and entorhinal cortex were chosen to reflect medial temporal area loss.

Total hippocampal and entorhinal cortex volumes were calculated by summing the data available from the left and right cerebral hemispheres. This bilateral involvement was used to examine overall medial temporal involvement.

All volumetric ROIs were normalized to each participant’s associated ICV to reduce confounding from differences in individual head and brain size, as shown in Equation (2):

(2) $$Normalize\;Volume\;=\;\frac{ROI}{ICV}$$

This produced the resulting variables hippocampus/ICV, entorhinal cortex/ICV, total GM/ICV, and cerebral WM/ICV.

Other measures were derived to investigate both global and regional relationships among the selected dependent variables. Brain parenchymal fraction (BPF) was calculated by summing the GM and WM while dividing by ICV (Equation (3)):

(3) $$BPF\;=\;\frac{Total\;GM\;+\;Cerebral\;WM}{ICV}$$

For local ratios, hippocampal fraction was calculated by dividing the hippocampal volume by Total GM (Equation (4)). This was utilized to see if the volume of the hippocampus changed relative to GM:

(4) $$Hippocampal\;Fraction\;=\;\frac{Hippocampus\;Volume}{Total\;GM}$$

Lastly, height, weight, and plasma tau217 levels were matched to the participant identity (PTID) to examine the relationship with BMI and a plasma biomarker.

### 2.3. Statistical Analysis

The volumetric parameters were mainly analyzed using mixed-effects models in Rstudio (2026.08.0 + 187). Linear mixed-effects models were fit using the lmer function from the lmerTest (version 3.2.1) and lme4 (version 2.0.6) packages with restricted maximum likelihood (REML). If an observation was missing in any variable, the data point was excluded, and no missing values were imputed. Model assumptions were evaluated with residual-versus-fitted, Q-Q plots, variance inflation factors, and convergence checks. Overall, the results supported the fitted model interpretation. The primary model included demographic variables like age, entry diagnosis, and sex as the fixed effects, with PTID as the random intercept. Genetic status, cognitive scores, and education status were not included as covariates because of unequal matching across the large cohort. PTID was used as a random intercept due to the presence of participants with multiple scans at different follow-up intervals, as these data points were interconnected. The main outcomes were the hippocampus, entorhinal cortex, GM, and WM normalized to ICV (ROI/ICV), as shown in Equation (5):

(5) ROI/ICV ~ Age_centered_ + Diagnosis + Sex + (1|PTID)

Variations in this equation and the interaction terms were explored for additional support. We also centered age to the mean and used CN and female sex as the reference categories. This means that entry diagnosis shows the adjusted difference between AD and CN, whereas sex represents the adjusted difference between males and females. Age, on the other hand, represents the expected change in ROI/ICV for each additional year of age based on the dataset.

Exponential models were fit for each diagnostic group for each of the anatomical variables. Equation (6) describes the overall age-associated percent decline observed in the cross-sectional data and estimates the associated annual percent change from the exponential rate coefficient rather than within-participant longitudinal atrophy rates:

(6) ROI/ICV = A × e ^(b×Age)^

To account for participants with multiple scans, the exponential decay parameters (A and b) and their standard errors were estimated using a participant-level cluster bootstrap with 1000 resamples, rather than treating each repeated scan as an independent observation. A nonlinear mixed-effects approach using MATLAB version R2026a (MathWorks, Natick, MA, USA) ’s nlmefit was also tested, but this did not converge reliably across all of the ROI and diagnosis combinations, so the cluster bootstrap was used instead as a more stable alternative. Scans with missing values for a given ROI were excluded from that ROI’s model, and this resulted in 12 to 73 scans being excluded per ROI and diagnostic group depending on the amount of missing data available.

Observational trends were incorporated to show how age, sex, and diagnosis-associated patterns appeared across each of the morphometric parameters. For these ROIs, the mixed-effects models were stratified by sex, and models were used to create expected trendlines for males vs. females. Each individual scan was plotted as a single point, and the lines were fit across the age range for each sex and diagnostic group. These were used to allow males and females to have their own age-associated slopes in order to visualize age-related trends as a linear function. Age bins were also created to summarize normalized volumes for 5-year age increments.

Body mass index (BMI) was analyzed separately as a predictor for the morphometric variables and also included age, diagnosis, and sex as fixed effects, while PTID remained the random intercept. This was used to examine whether BMI had its own association with each of the ROI volumes and whether these relationships were different for CN vs. AD. Plasma tau217 levels were matched to each of the PTIDs at their associated MRI date. These biomarker data were more limited compared to the much larger MRI volume dataset. Therefore, the plasma-based biomarker was used as a secondary, exploratory approach. The Spearman correlation for BMI and pTau217 were descripted analyses, and the *p*-values were not adjusted for multiple comparisons.

## 3. Results

### 3.1. Cohort Characteristics

The data analyzed included repeated 3T MRI scans from both CN- and AD-diagnosed participants. The number of scans and participants varied slightly for each ROI because not every scan had each morphometric variable available, possibly due to segmentation or data collection errors. Each morphometric parameter and associated number of scans and participants are summarized in Table 1. Overall, there tended to be more female than male participants and scans, with overall total participants varying only slightly among the different ROIs.

**Figure 1:**
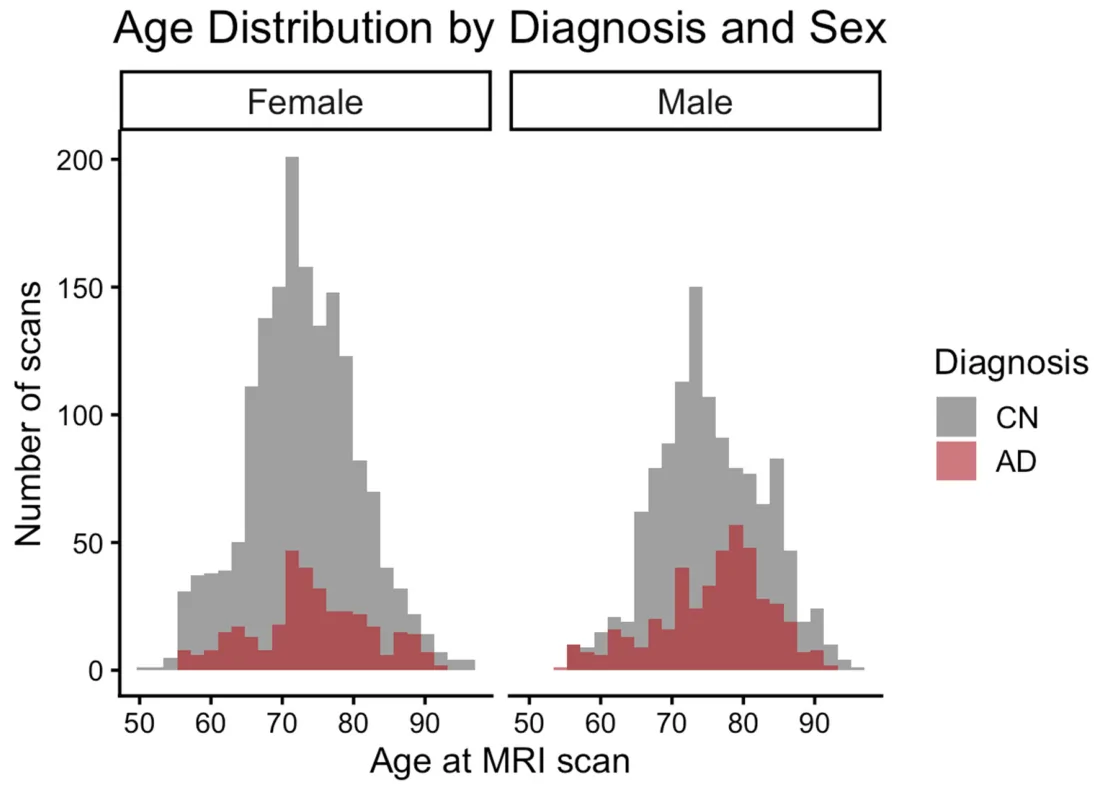
Distribution of age by diagnosis and sex. The histogram plot shows the number of scans in each diagnosis group (AD and CN) for males and females. Each observation is representative of a single MRI scan, with repeated scans of participants included for each MRI visit. Fewer data points were available at both extreme ends of age.

Figure 1 shows that there were more CN than AD scans overall, and that most scans were concentrated between the ages of 65 and 85. Furthermore, both ends of the age spectrum have a lower number of scans, which could reflect the typical age of AD onset and survivorship bias in older ages.

### 3.2. Linear Mixed-Effects Models

Linear mixed-effects modeling was used to examine the association between age, diagnosis, sex, and morphometric parameters normalized to ICV. Repeated scans from the same participant were accounted for by including PTID as a random intercept. Table 2 explores the model results. Older age was associated with lower normalized volume for each morphometric parameter, and AD diagnosis was also associated with a lower normalized volume after adjusting for age, sex, and repeated scans. Lastly, sex was also associated with differences in regional volume, although the effects were smaller and the direction varied by ROI.

**Figure 2:**
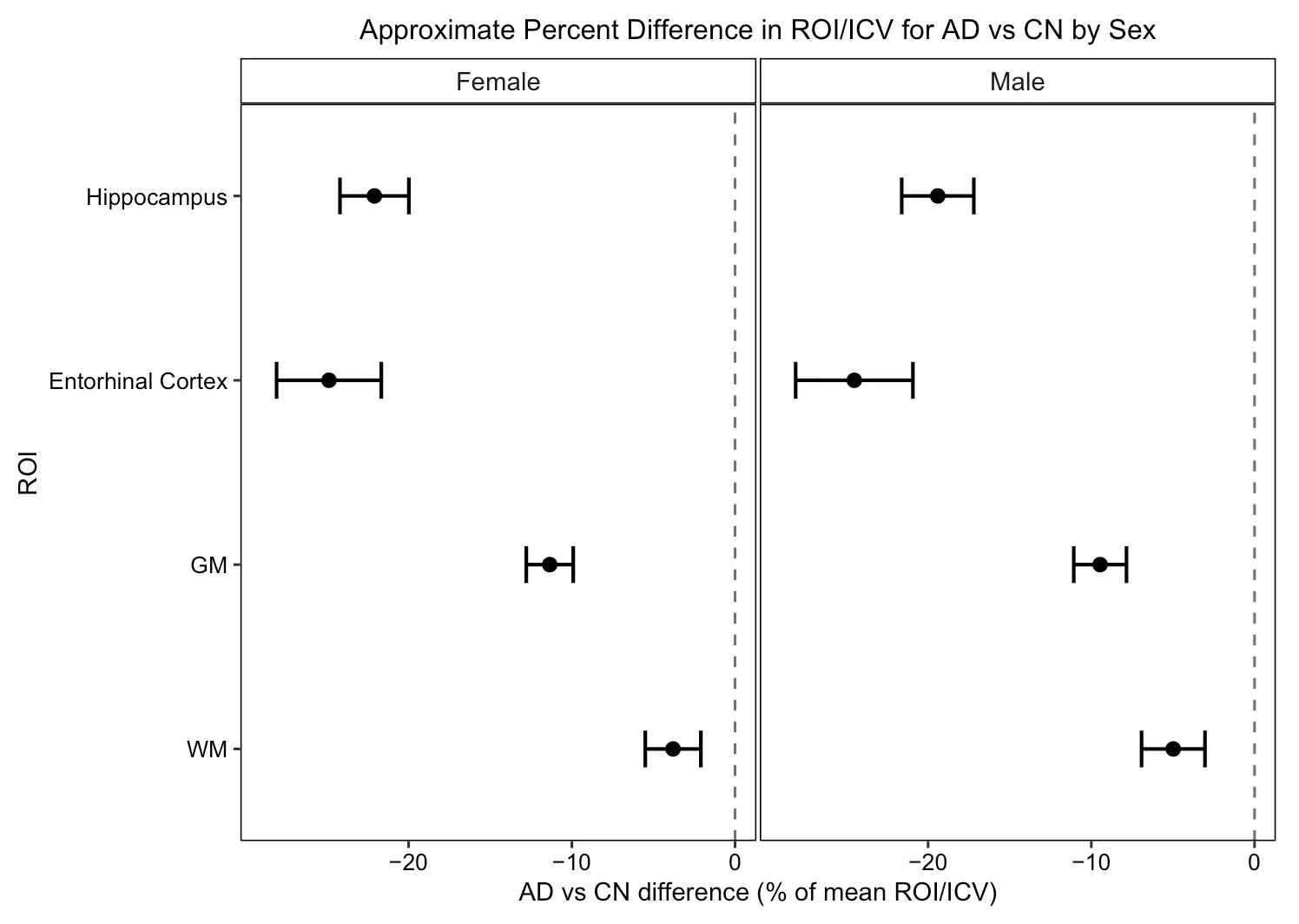
AD vs. CN adjusted differences for morphometric variables separated by sex. Each point represents the adjusted AD vs. CN value from the sex-stratified linear mixed-effects model, while the error bars represent 95% confidence intervals. The model incorporated mean-centered age and diagnosis for the fixed effects and PTID as a random intercept for each sex of the form [ROI/ICV ~ Age_centered_ + Diagnosis + (1|PTID)]. Values are expressed as a percentage of the mean ROI/ICV for the region of interest.

Relative to the mean ROI/ICV value, the AD-associated differences were greatest for the medial temporal regions (hippocampus and entorhinal cortex). AD participants showed a lower normalized volume for each ROI in both males and females alike. When the diagnosis × sex interaction was analyzed, there was no significant interaction. This suggests that the relative volume separation between AD and CN was comparable among males and females, as supported by Figure 2. In Figure 2, we see that the entorhinal cortex had the largest percent difference when comparing AD and CN participants. AD males and females showed 24.6% and 24.8% lower volumes compared to the CN group, respectively. Comparatively, normalized hippocampal volumes were about 20.3% lower in AD males and 21.3% lower in AD females when compared to CN participants.

Figure 2 shows that among the global parameters, GM had a greater degree of separation between AD and CN participants. AD females showed an 11.2% lower GM volume compared to CN females. Similarly, AD males showed a 9.68% lower GM volume compared to their CN counterparts. For cerebral WM, there was the smallest relative difference. Specifically, AD females and males showed a 3.8% and 4.9% difference compared to CN participants, respectively.

### 3.3. Volume Trends Across Groups

**Figure 3:**
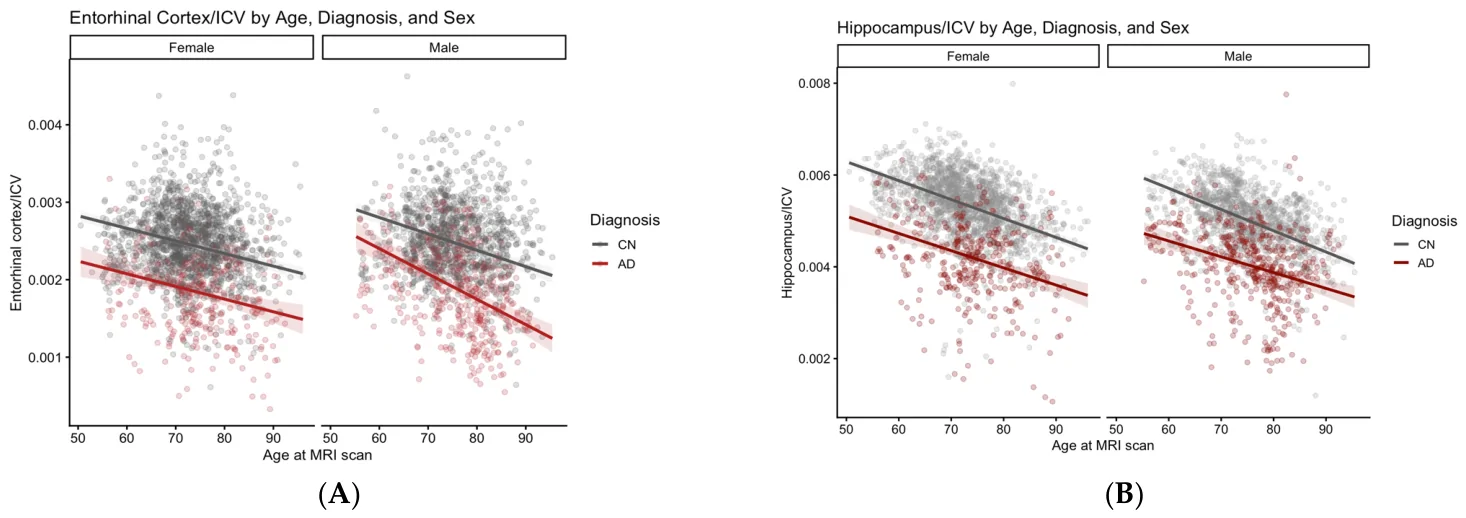
Entorhinal cortex (**A**) and hippocampus (**B**) normalized to ICV by age, diagnosis, and sex. Each individual point represents a single MRI scan at the associated age of the participant. The solid gray line represents the CN group, whereas red represents the AD group. Lines represent the model-predicted values from the linear mixed-effects model with PTID as a random intercept. Shaded bands are 95% CIs.

Across the data for the hippocampus and entorhinal cortex, AD participants showed, on average, lower regional volume loss when normalized to ICV. For the hippocampus in Figure 3, AD participants had a lower normalized hippocampus at older ages. This was consistent for males and females alike, as the separation was consistent with AD participants showing lower normalized volumes across the observed age range. The entorhinal cortex showed a similar pattern, which also supports the previous model in which medial temporal areas were lower for AD vs. CN groups.

**Figure 4:**
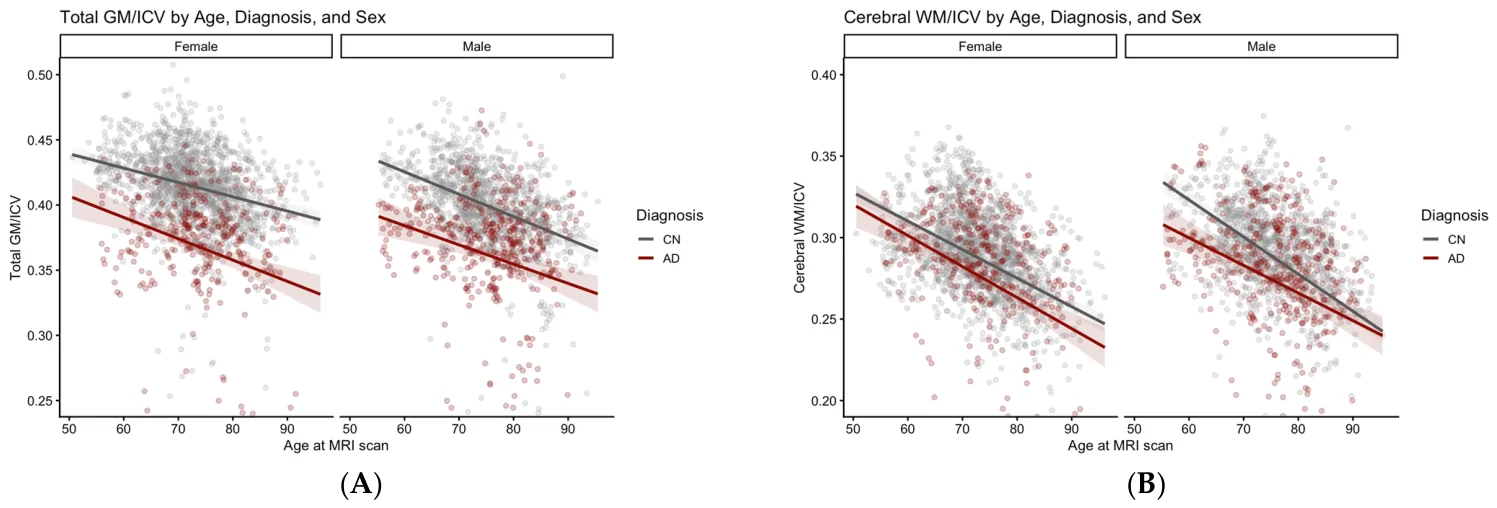
Total gray matter (**A**) and cerebral white matter (**B**) normalized to ICV by age, diagnosis, and sex. Each individual point represents a single MRI scan where gray and red represent CN and AD groups, respectively. Lines represent the model-predicted values from the linear mixed-effects model separated by sex, with 95% CI shaded bands.

The global tissue parameters had a similar age-associated trend in the negative direction, as seen in Figure 4. Total GM decreased with age in both males and females, with AD participants showing lower normalized volume loss compared to CN individuals. Cerebral WM also had a negative slope for each diagnostic group and sex, but the separation was not as prominent. When compared with the medial temporal regions, the downward shift was not as prominent.

These plots can help support the other results by showing that older age and diagnosis are the major variables associated with lower normalized volume for each of the four ROIs. This visual pattern can also suggest that age-associated trends vary by anatomical morphometry, sex, and diagnosis. These plots should be interpreted as descriptive using the sex-stratified mixed-effects model and not as direct estimates of individual disease progression.

**Figure 5:**
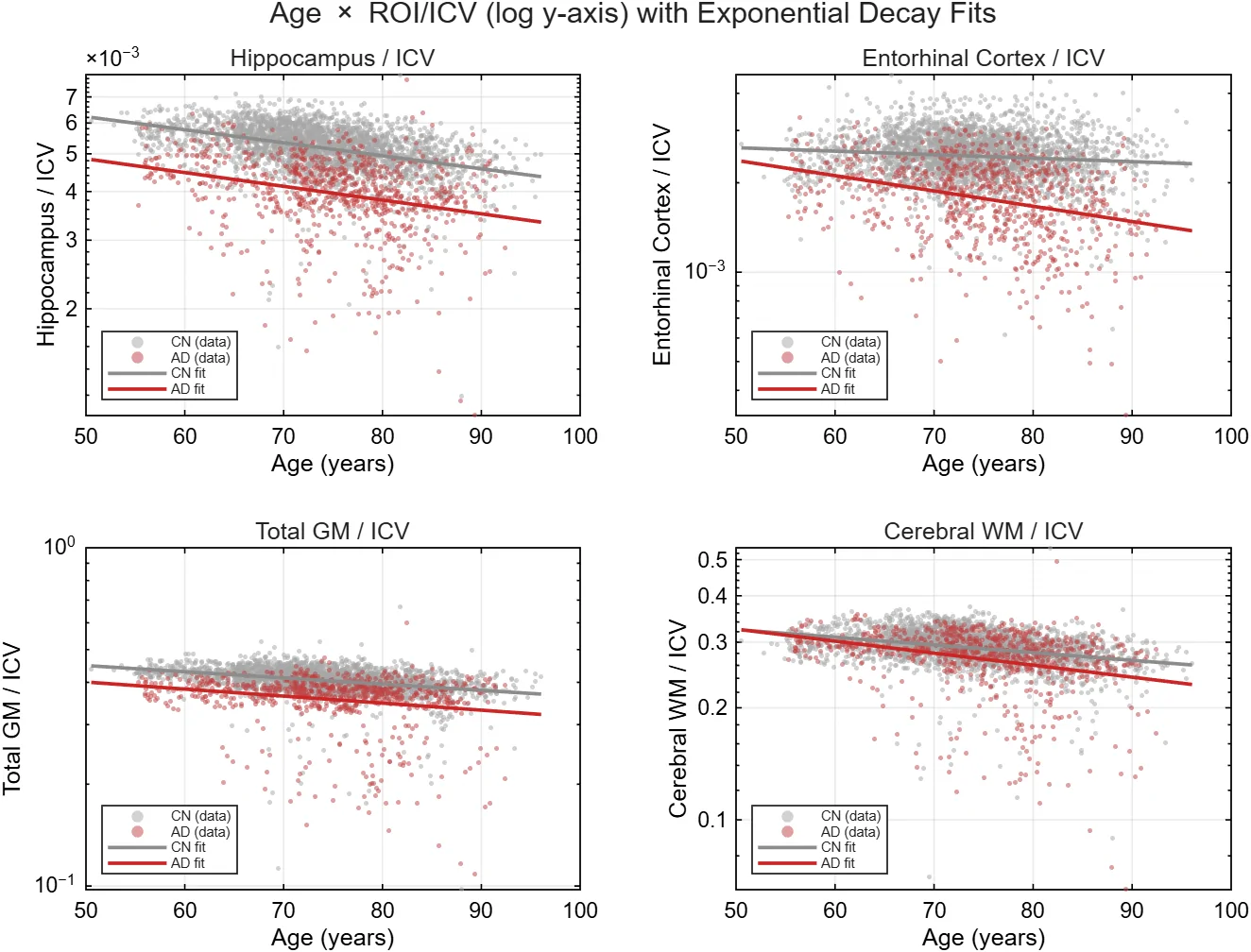
Log-scale exponential decay fits for age-associated ROI relationships by diagnosis. Scatters show individual MRI scans for CN and AD participants for each ROI. Solid lines are the exponential model fits by diagnostic group (AD, red; CN, gray). The *y*-axis is shown as a logarithmic scale to visualize the exponential form of the age and volume relationship.

Exponential decay models of the form Volume/ICV = A × e^(b×Age)^, with b being a rate-of-change parameter as a function of age, were introduced in Figure 5 to obtain an alternate mathematical description of the relationship between brain volume and age and to estimate the approximate amount of decrease by age for each region and diagnostic group. This estimates the percent annual decrease directly from the model coefficient, with the age of the subject as a parameter, and is presented in Figure 5. The equations for each ROI for the CN and AD groups are summarized in Table 3, and the percent difference in the ROI/ICV estimated from them as a function of age are summarized in Table 4.

These exponential models fit the cross-sectional age–brain volume relationship in this dataset. They should not be interpreted as accurate estimates of individual participants’ true longitudinal atrophy rates, as they are not continuous within-person trajectories. The CN group’s hippocampus (−0.73%/year), cerebral WM (−0.45%), and total GM (−0.40%) were the most age-sensitive areas, as was the entorhinal cortex (−0.22%), which is consistent with results from the mixed-effects analysis in Section 3.2.

The age-associated percent decline for AD was greater in magnitude compared to CN throughout the regions. The largest separation was in the entorhinal cortex (AD −1.00%/year vs. CN −0.22%/year), whereas the separation in the hippocampus was more moderate (AD −0.77%/year vs. CN −0.73%/year). For total GM, the CN group showed a marginally larger decline than the AD group (CN −0.40%/year vs. AD −0.38%/year), while cerebral WM showed the opposite pattern, with a larger decline in AD (−0.62%/year) than CN (−0.45%/year). Put together, these findings argue that, beyond the group-level analysis, the largest AD-associated deviation from the CN trajectory is located in the entorhinal cortex, rather than just in the hippocampus.

**Figure 6:**
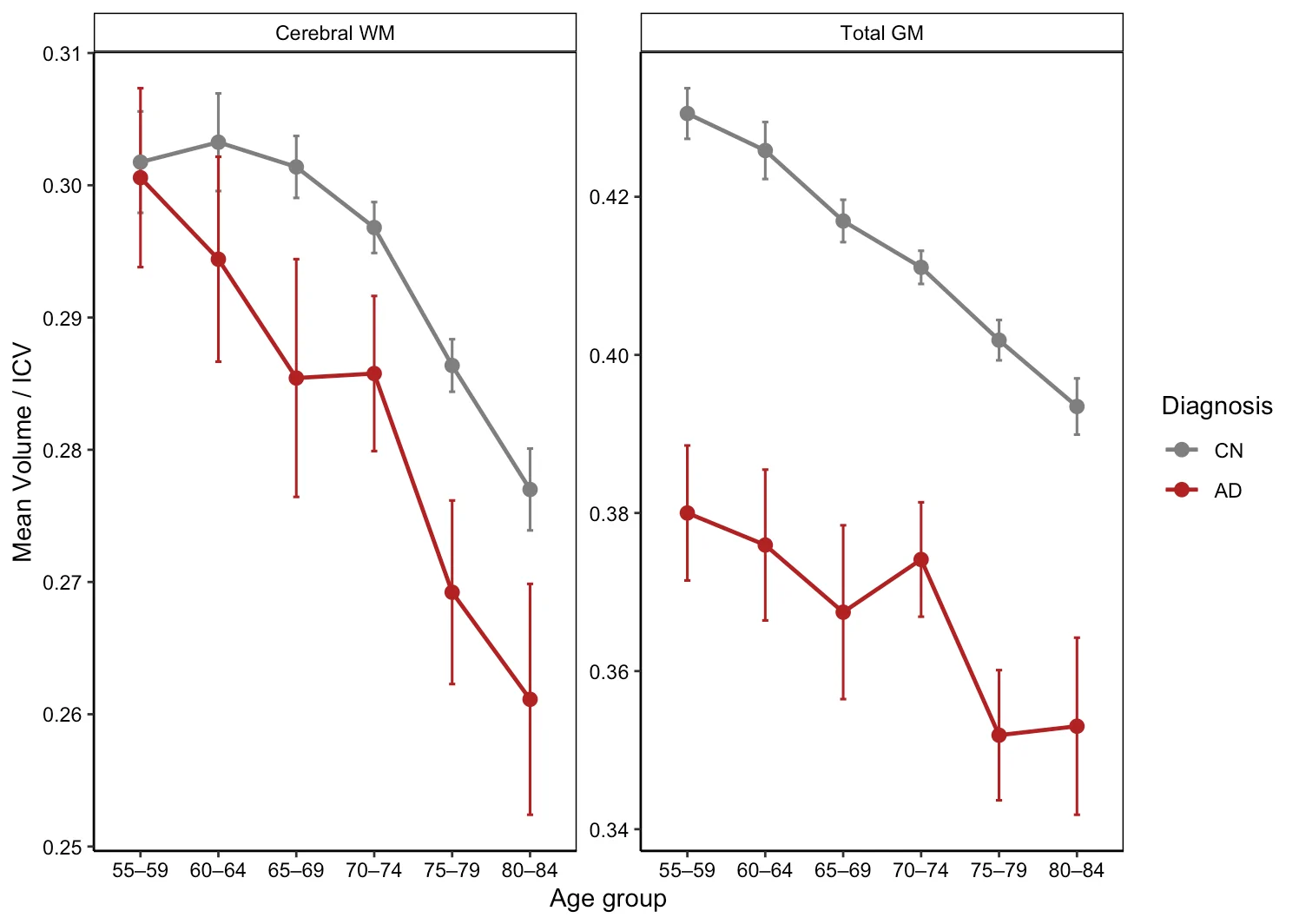
Mean GM and WM volume normalized by ICV by age group and diagnosis. Points represent the mean normalized volume at each age group, while the bars represent the 95% confidence intervals. CN participants had a smoother age-associated decline, while AD participants had greater variability and CI bars. The degree of separation is greater in GM compared to WM.

To add to the mixed-effects model and above scatters, the mean volumes for the global parameters (GM and WM) were plotted based on 5-year age groups. Figure 6 shows the age-related decline in GM and WM volume for each diagnostic group. Overall, the CN participants showed a generally smoother decline in volume compared to their AD counterparts. The greater variability within the AD data points could be due to smaller available scan number and heterogeneity in AD severity (mild, moderate, and severe).

**Figure 7:**
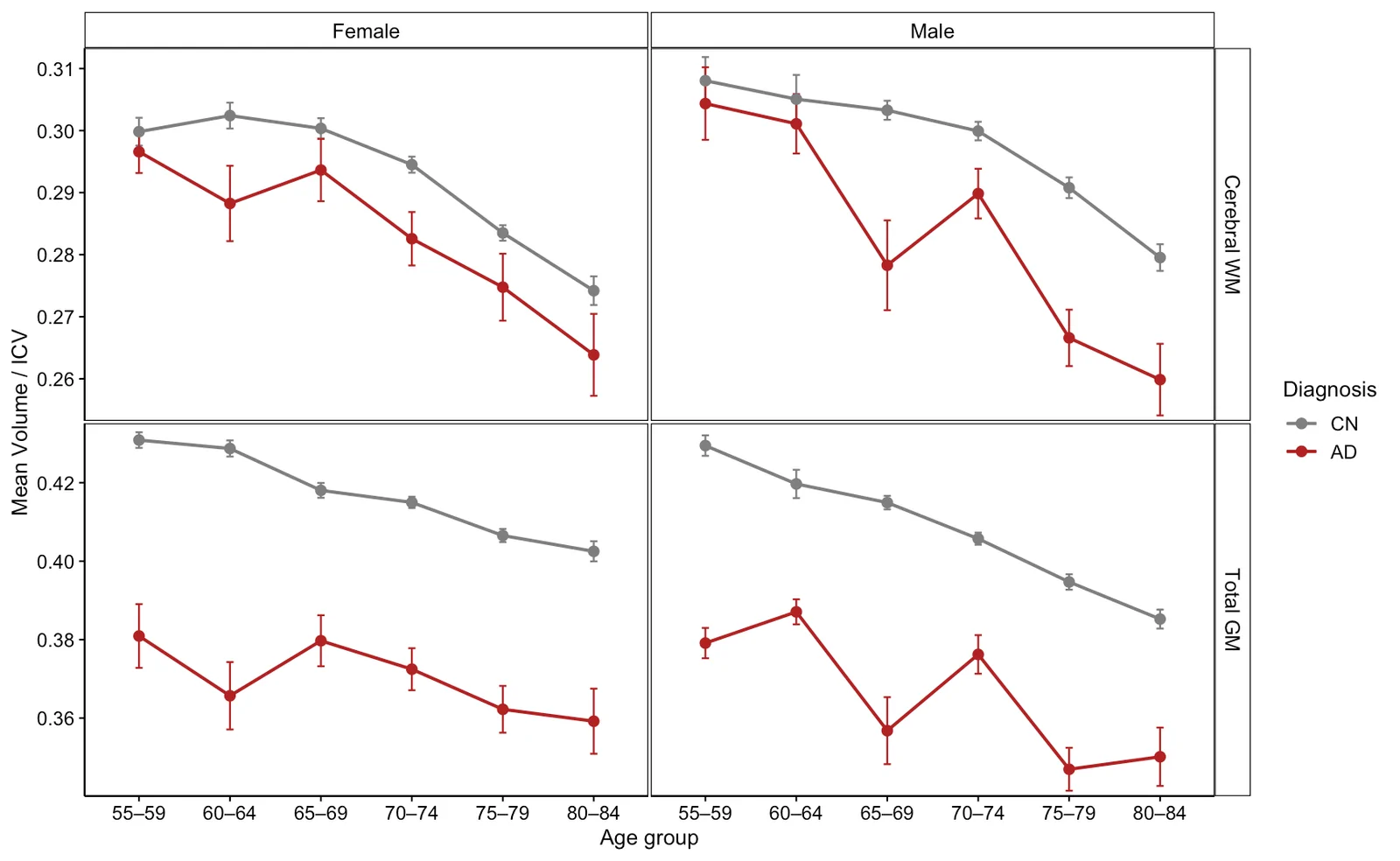
Mean total GM and WM normalized to ICV by 5-year age groups, diagnosis, and sex. Points represent the mean at each age group, and error bars are the 95% confidence intervals. For both males and females, there was an overall decline in volume, and the degree of separation is greater for AD participants compared to CN.

When the data is split by sex, AD males and females both showed a lower mean GM and WM volume when normalized to ICV. The separation was more pronounced in the GM dataset and remained consistent with the mixed-effects model. For the male dataset, there appears to be a large decline at ages 65–69 followed by an increase at 70–74 years (Figure 7). This pattern does not likely reflect the true population of AD participants but rather the available volumetric data. A relative increase in volume is not consistent with AD pathology, and thus the overall negative decline should be emphasized. Like the above findings, the degree of separation was more prominent in GM. In general, the AD group had higher confidence intervals. This greater uncertainty around age bins is likely due to differences in individual AD progression and disease pathology.

**Figure 8:**
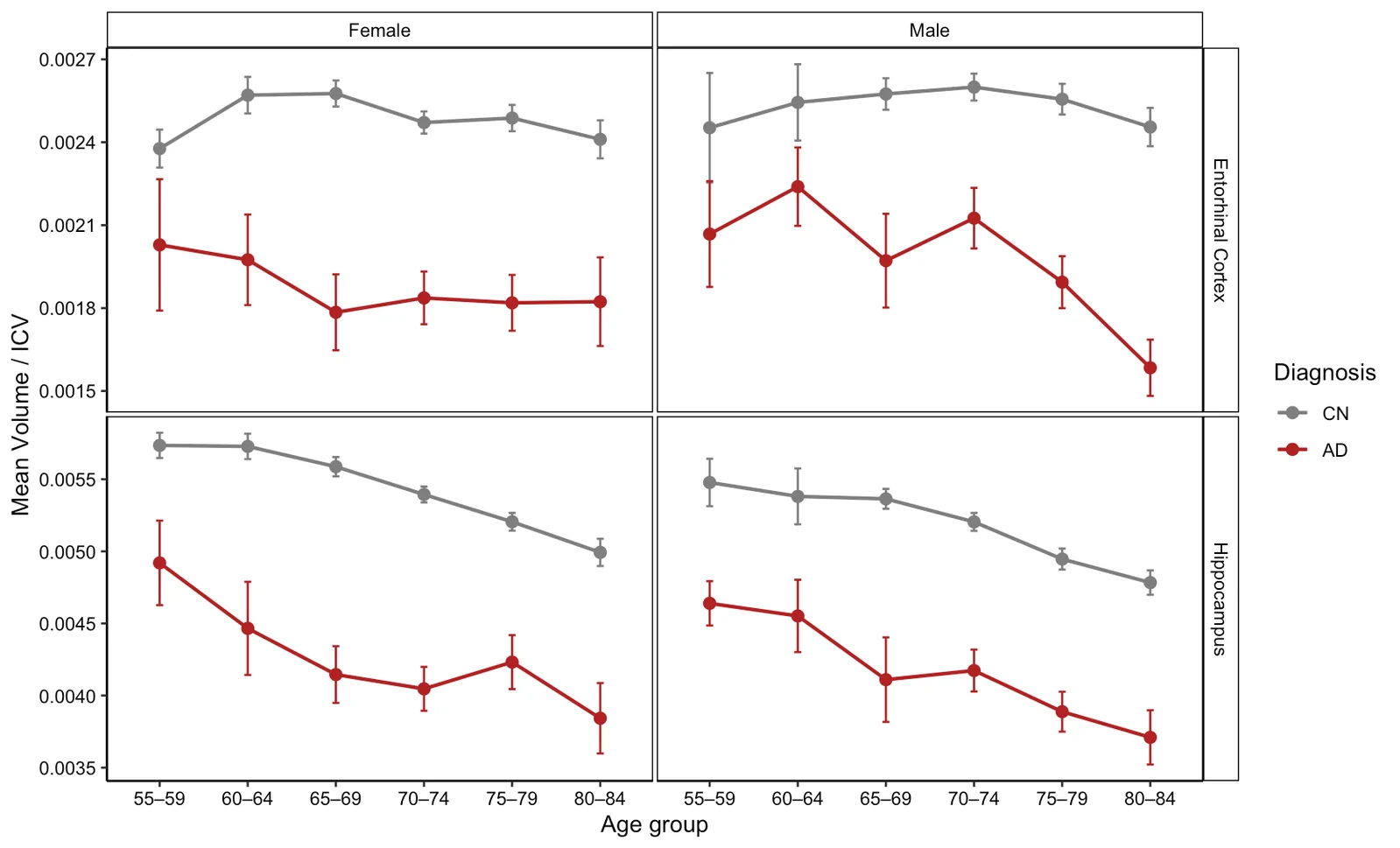
Mean entorhinal and hippocampal volume normalized to ICV by 5-year age groups, diagnosis, and sex. Points represent the mean at each age group, and error bars are the 95% confidence intervals. For both males and females, there was an overall decline in volume, and the degree of separation is greater for AD participants compared to CN.

**Figure 9:**
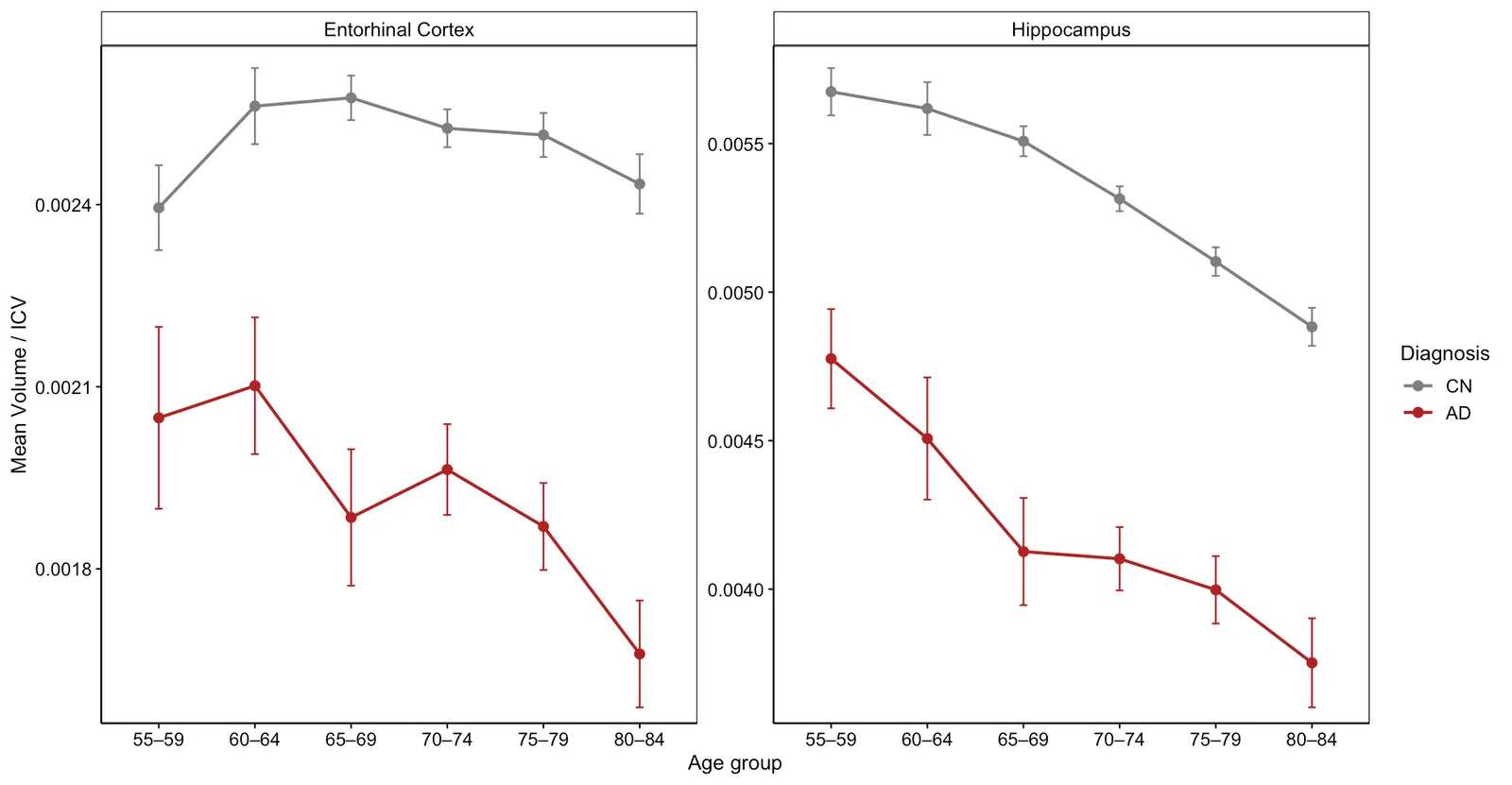
Mean entorhinal and hippocampal volume normalized to ICV by age group and diagnosis. Points represent the mean normalized volume at each age group, while the bars represent the 95% confidence intervals. CN participants had a smoother age-associated decline, while AD participants had greater variability and wider CIs.

Mean hippocampal and entorhinal volumes demonstrated clearer separation across diagnostic and age groups. As seen in Figure 8, AD participants had lower overall volumes with a greater degree of uncertainty around the mean for both males and females. The CN group showed smoother age-associated decreases. Males and females showed an overall similar negative trend with age, with minor increases and decreases likely due to individual heterogeneity and cross-sectional sample size. Local increases are likely not due to true volume improvements for males and females at the associated age groups. These overall patterns were similar when males and females were combined, as shown in Figure 9.

### 3.4. Relationship with Body Mass Index (BMI)

A linear mixed-effects model was used to see the relationship between BMI and normalized ICV brain volumes. Fitted lines were plotted for the calculated BMI and associated normalized ROI volume. The BMI and the BMI × Diagnosis were found to be nonsignificant for each of the regions. This suggests that the BMI–volume relationship did not significantly differ between AD and CN groups.

For each ROI, AD participants generally had a lower ICV-normalized volume compared to CN participants. In terms of the global GM and WM parameters, the separation was more apparent for GM. The hippocampus and entorhinal cortex had a similar degree of separation compared to the global GM and WM plots. Spearman correlation did not support a strong association between BMI and the morphometric variables. White matter showed ρ = 0.009 and ρ = −0.023 for CN and AD participants, respectively. GM showed ρ = 0.026 for CN and ρ = −0.016 for AD participants.

**Figure 10:**
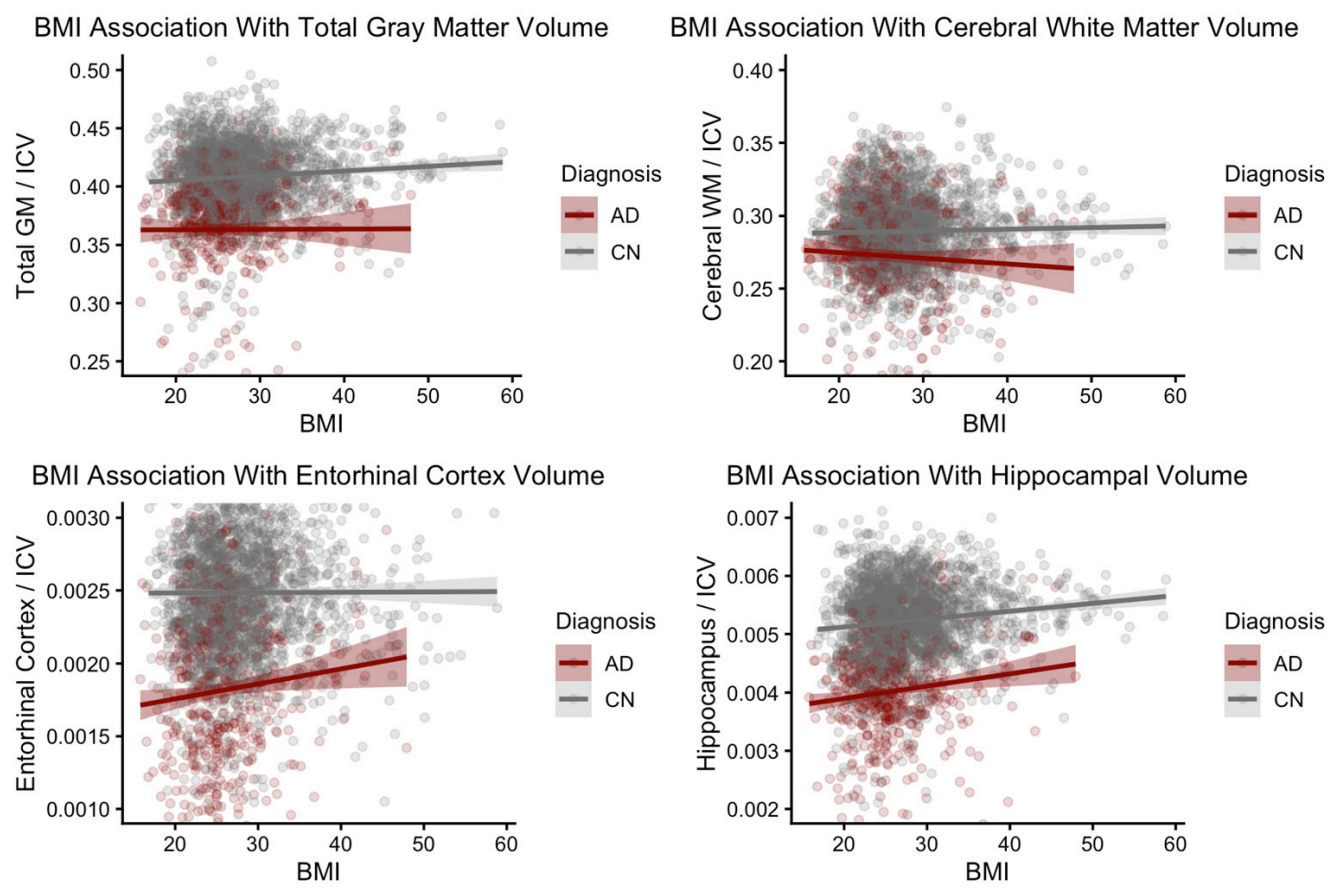
BMI relationship with ROI normalized brain volume. AD participants had overall lower normalized brain volumes compared to CN. Each point represents one MRI scan. The fitted lines show the trends by diagnosis, with a shaded 95% confidence interval. AD group had lower normalized volumes overall. The BMI slopes were shallow and nonsignificant.

On the other hand, the medial temporal areas had slightly stronger and positive associations, as seen in Figure 10. The hippocampus had a weak positive association with CN and AD (ρ = 0.108, *p* < 0.001; ρ = 0.113, *p* = 0.012). The entorhinal cortex showed a negligible positive association with BMI, with CN participants (ρ = 0.029, *p* = 0.17) and AD participants (ρ = 0.093, *p* = 0.039). However, these are descriptive statistics and do not account for age and sex as the initial model did. It is important to note that some of these findings could reflect a lower number of data points in higher BMIs in the AD group.

### 3.5. Diagnosis-Dependent Relationships Among Global and Medial Temporal Structures

**Figure 11:**
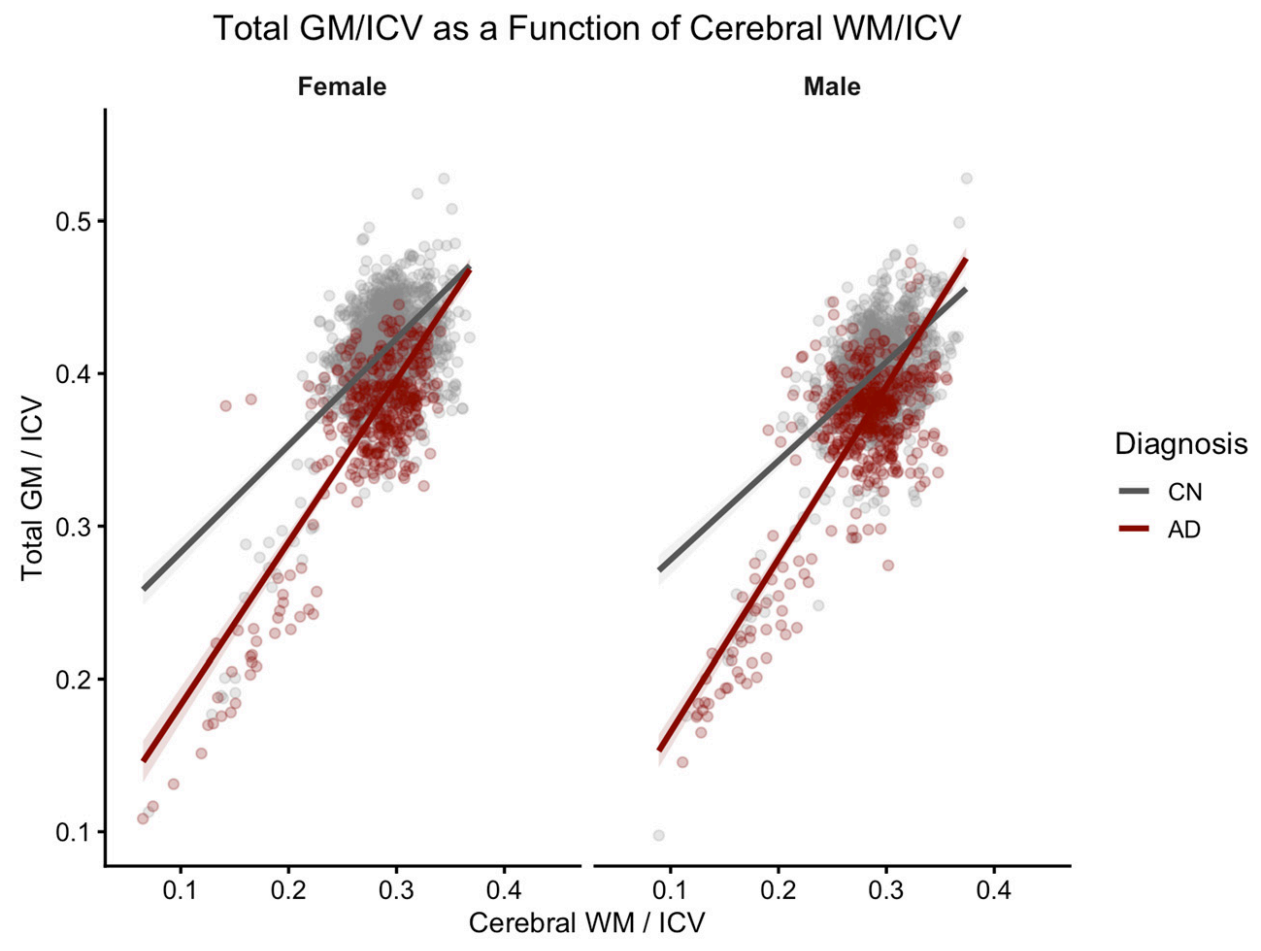
Relationship between cerebral WM and Total GM when normalized to ICV. Shaded 95% CI. The plot shows that GM and WM are positively related (as expected) in both CN and AD. The AD case appears to shift toward lower GM relative to CN. In other words, for a similar amount of normalized cerebral WM, AD subjects could have lower normalized GM than CN people.

We further examined the ROIs in relation to global and local ratios, where GM was a function of WM and hippocampus was a function of entorhinal cortex. This was used to determine if AD and CN participants differed in global and medial temporal relationships. Total GM was positively associated with WM across groups. Table 5 summarizes how white matter was significantly associated with GM in the CN reference group. Additionally, the cerebral WM × diagnosis interaction was also significant, which supports that the relationship between WM and GM differed by diagnostic group. In Figure 11, AD and CN both show positive GM-WM trends, although AD participants shifted toward a lower GM relative to CN at a similar WM level. Figure 11 also suggests that the male and female differences are similar and that the overall GM-WM association is consistent across sex for AD and CN groups alike.

**Figure 12:**
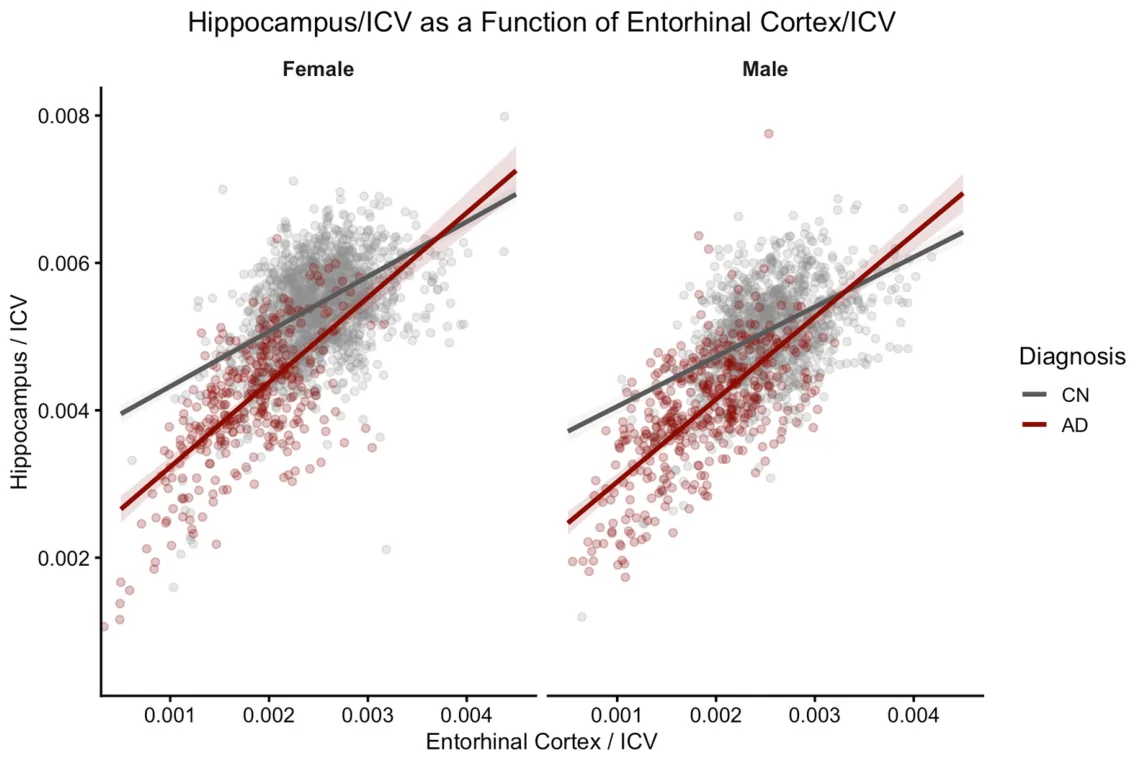
Scatterplot of the relationship between entorhinal cortex and hippocampus normalized to ICV. Males on the right and females on the left. These medial temporal regions are known to be influenced by AD volumetric decline.

Figure 12 supports that medial temporal regions showed a comparable pattern for both males and females alike. As supported in Table 5, ICV-normalized hippocampal volume was positively associated with the entorhinal cortex for the CN reference group. Furthermore, Table 5 shows that the entorhinal × diagnosis interaction was also significant. This statistically supports that the relationship between the hippocampus and entorhinal cortex differed by AD and CN groups. Figure 12 shows that males and females showed comparable positively associated trends for CN and AD alike, with the AD fitted line being steeper and shifted compared to CN. This suggests that this medial temporal relationship is not parallel across diagnostic groups. In other words, for a given entorhinal cortex volume, hippocampal volume will scale differently for AD vs. CN participants.

**Figure 13:**
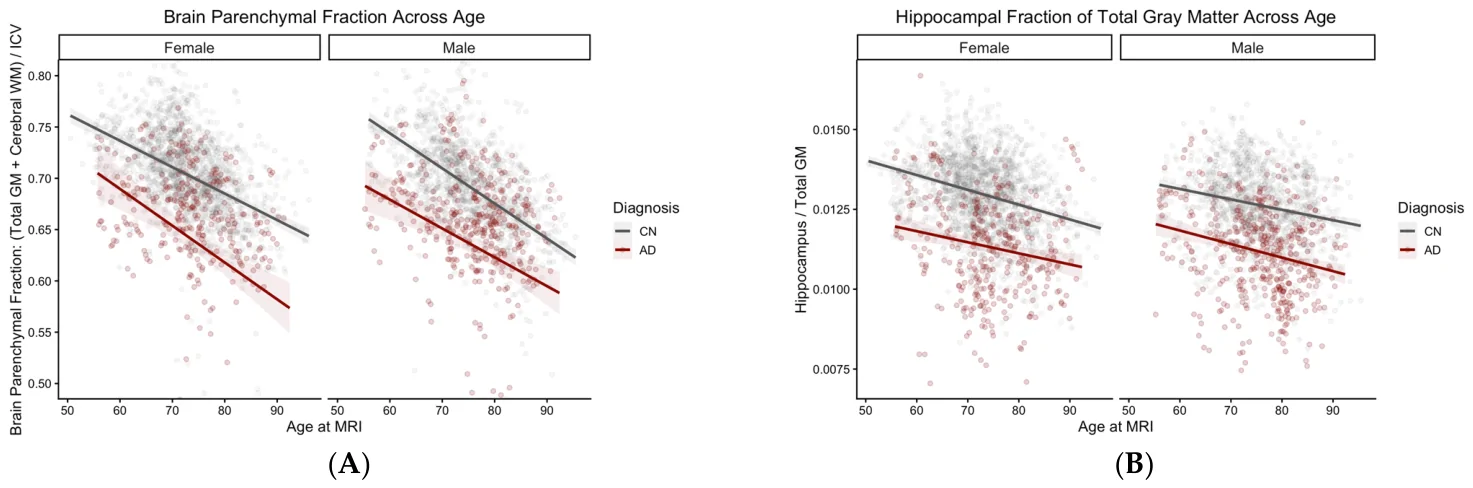
Global and local ratios across age, diagnosis, and sex. (**A**) BPF as a function of age at the time of an MRI scan. (**B**) Hippocampal fraction as a function of age at MRI scan. Both ratios show a negative association with age for both males and females, with an overall lower ratio for the AD group. AD and CN slopes were broadly comparable across plots due to diverse dataset and patient heterogeneity. Shaded regions represent the 95% CI of the fitted trendline.

Brain parenchymal fraction (BPF) was calculated by summing the total GM and cerebral WM divided by ICV. The data show that this fraction decreases in both AD and CN participants for both males and females (Figure 13). Across the cohort’s age range, AD participants had a lower overall BPF than CN, which indicates that these subjects had lower brain tissue volume when relating to the intracranial space.

The hippocampal fraction of total GM was also calculated by dividing the hippocampal volume by total GM. Figure 13 shows the negative relationship of hippocampal fraction with age. AD participants had an overall lower hippocampal fraction compared to the CN group for both males and females alike. In other words, this supports that the hippocampus is not simply reduced because of overall GM loss, but rather is disproportionally reduced in AD. The minor slope deviations for CN and AD groups are likely due to a diverse dataset and an uneven number of scans for each diagnostic group but, overall, are comparable for these brain volumetric parameters.

### 3.6. Plasma Phosphorylated Tau Association with Volume

Plasma tau217 concentrations were generally higher in the AD group compared to CN. This analysis included 289 observations from 243 unique individuals (140 female, 103 male, 202 CN, and 41 AD). Because of the great reduction in sample size and not being adjusted for diagnosis, age, and sex, plasma pTau217 analysis should be viewed as exploratory.

**Figure 14:**
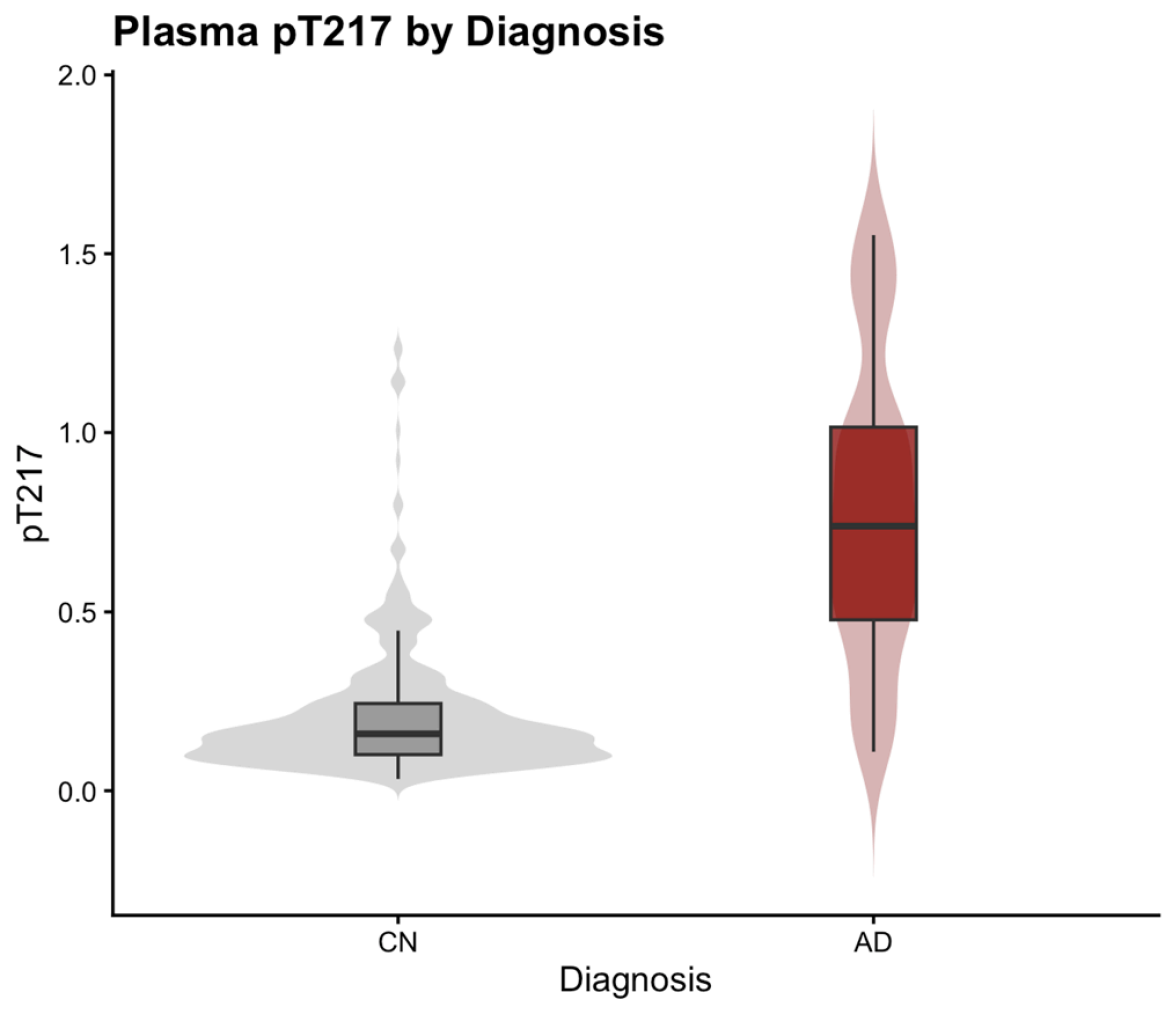
Distribution of phosphorylated tau217 for CN and AD participants. Violin plots show the overall distribution of phosphorylated tau217 for CN and AD groups. In general, AD participants had a higher median pT217 with greater variability.

**Figure 15:**
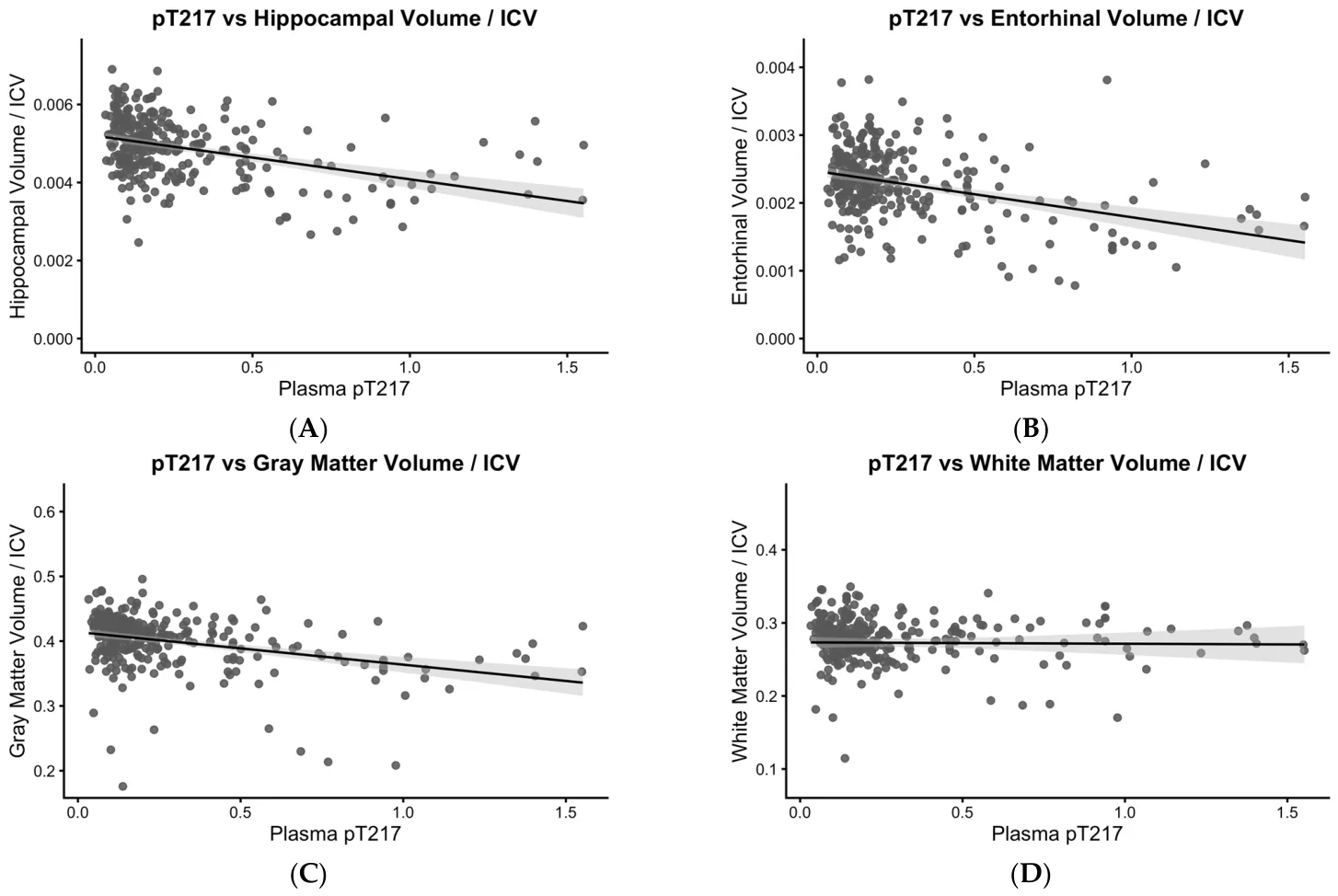
Plasma pT217 association with each ICV-normalized volume. Each scatterplot shows pT217 plotted against (**A**) hippocampus, (**B**) entorhinal cortex, (**C**) total GM, and (**D**) cerebral WM. Fitted lines represent linear trends with 95% confidence interval bands. In general, higher pT217 values were associated with lower entorhinal, hippocampal, and GM values, while WM showed little association.

The median AD pT217 was about 4.6 times greater than CN (AD: 0.70 pg/mL, IQR, 0.47 to 0.87 pg/mL; CN: 0.15 pg/mL, IQR, 0.10 to 0.25 pg/mL) and had a broader distribution of values, as seen in the violin plot for Figure 14. The group difference was statistically significant with a Wilcoxon rank-sum test (*p* < 0.05). This provides context for fitted plots seen in Figure 15, where higher pT217 concentrations correspond with lower normalized volumes in the entorhinal cortex, hippocampus, and GM. The correlations observed may reflect between-group separation and should not be explained as diagnosis-independent or within-diagnosis associations. This focus was on the relationship between the concentration of the biomarker and MRI-derived volume.

The pooled correlations with tau and MRI volumes were negative across each regional measure. There was the strongest association with the hippocampus, followed by total GM and entorhinal cortex, as summarized in Table 6. Cerebral white matter was just barely found to be statistically significant and showed the weakest association.

## 4. Discussion

### 4.1. Summary of Main Findings

In this study, we used 3T MRI data to describe ICV-normalized brain volumes in both Alzheimer’s disease and cognitively normal participants. Aside from confirming lower volumes in people with AD, this contribution shows that the magnitude and proportional relationships of morphological regions vary across global and medial temporal structures within a broad cohort. The mixed-effects modeling, direct global-versus-regional comparisons, and derived morphometric fractions allowed us to show the relative and disproportionate tissue loss between each group (AD vs. CN).

After adjusting for age, sex, and repeated scans, AD participants showed significantly lower normalized volumes compared to the CN participants. The biggest AD-related differences were seen in the medial temporal regions. In particular, the AD group had roughly 25% lower normalized entorhinal cortex volume and around 20–21% lower normalized hippocampal volume than the CN participants for both females and males.

When we looked at global measures, both GM and WM decreased with age. However, the separation between AD and CN groups for both males and females showed more difference in GM than for WM. Although the sex-related differences were observed, they were smaller in magnitude and less consistent than the effects linked to age and diagnosis. Regarding BMI, the data did not suggest it played a major role in brain volume associations in this cohort.

For the systemic biomarker, plasma pT217 was higher in AD participants compared to CN participants. It also showed a negative relation with the hippocampus, entorhinal cortex, and total GM volumes, with the strongest relation found in the hippocampus. Overall, these results support the idea of structural MRI morphometry, especially within the medial temporal lobe, and connect to disease-related atrophy in AD. This suggests that the MRI-based structural measures may be a useful complement to blood-based biomarkers.

### 4.2. Age and Alzheimer’s Disease Effects on Brain Morphometry

#### 4.2.1. Age-Related Brain Volume Changes

One of the most consistent findings across our dataset for the regions analyzed was that there was a negative association between age and ICV-normalized brain volumes, regardless of diagnosis or sex. This effect was observed in the CN and AD groups and remained throughout the age range covered in the study. Brain volume loss has been known to occur with normal aging and neurodegenerative disorders and can be widespread [22,23]. This encompasses a decrease in the volume of multiple brain regions, even in the absence of a neurodegenerative disease [24,25]. Longitudinal and meta-analysis neuroimaging studies have reported that, with aging, cognitively normal adults lose around 1% of their hippocampal volume annually compared to 3–5% in MCI and AD patients, and that this rate of hippocampal volume loss begins to occur in a measurable way in middle age [26,27,28]. The atrophy rates presented from prior studies are longitudinal, and thus not comparable to the cross-sectional, model-based rates reported in the present study, which reflect between-person age differences rather than within-person change over time. Our findings are also consistent with the existence of clear group-level differences across a large MRI sample, even though we are of a retrospective design with limited ability to estimate atrophy within persons annually. The observed separation between AD and CN remained consistent after adjusting for age, sex, and repeated scans, supporting the value of a cross-sectional, repeated-measures approach for detecting group-level differences, independent of the longitudinal atrophy-rate estimates discussed above.

In line with these results, VBM studies have demonstrated that a loss of gray matter volume and associated brain atrophy was more prominent in dementia groups compared to cognitive health, with emphasis on degeneration of medial temporal structures [29]. Widespread atrophy is also noted in both gray and white matter changes but may be to a more limited extent in a somewhat different distribution [22,29]. Our study population supports this progressive brain volume loss with age in each of the four ROIs and can provide a common reference against additional effects of AD diagnosis.

Meanwhile, this baseline trend is important to consider, as it provides the context to understand the extra loss of volume in the AD group. In their review, Woodward et al. found that the rate of hippocampal atrophy in AD patients is around 3–5 times higher than that of cognitively unimpaired older adults. As per the report, in normal controls, there will be a loss of 1% per year, while in AD, there will be a loss of approximately 3–5% per year [27]. This is in line with the hypothesis that the hippocampus is quantitatively but not categorically impaired. These figures reflect longitudinal, within-subject measurements and are cited here as background context rather than as a benchmark directly tested in our cross-sectional dataset.

AD’s plasma biomarkers are associated with neuronal loss that affects specific brain regions [30,31]. As a result, disease-related atrophy creates volumetric differences that are detectable on structural MRI, and these changes differ from what is typically observed in normal aging [25,32]. In our study, AD participants had significantly lower normalized brain volumes than CN participants even after adjusting for sex and age. This suggests that the differences between groups in diagnosis are not solely attributable to structural aging.

#### 4.2.2. Medial Temporal Lobe Atrophy in Alzheimer’s Disease

Within this cohort, the medial temporal lobe structures exhibited the biggest AD-related differences. Their functions resemble those of the clinical profile of AD, and these regions play a crucial role in memory formation, spatial navigation, and early encoding of new information [33,34,35]. Loss of hippocampal volume is one of the first and most severely affected structures in AD and can be detected even in the preclinical stage [36,37]. This was confirmed by Hua et al. in a large sample of 676 participants [38]. Using tensor-based morphometry, they found strong bilateral hippocampal and temporal lobe atrophy in AD compared to controls. Previous studies have also validated the ICV-normalized hippocampal volume as a sensitive marker of disease severity and as a predictor of conversion from MCI to AD [36].

#### 4.2.3. Hippocampal Vulnerability and Subfield Degeneration

The size differences in the hippocampus seen in our data are comparable to earlier studies and sustain the significance of this region in the context of AD-related pathology. From ICV-normalized volumes of the hippocampal subfields in amyloid-confirmed AD-spectrum participants, Christopher-Hayes found that subfield volumes in the CA1, dentate gyrus, and subiculum were significantly reduced in amyloid-confirmed AD participants compared to amyloid-negative participants [39]. They studied the ICV-normalized volumes of subfields within the hippocampus in amyloid-confirmed AD spectrum participants and reported that there was a significant difference in subfield volumes between the dentate gyrus, CA1, and subiculum in amyloid-confirmed AD spectrum participants compared to amyloid-negative controls. They also showed recollection and lower MMSE scores. Their findings indicate that hippocampal volume loss in AD is not a generalized decrease but rather a selective vulnerability of specific subfields of the hippocampus involved in encoding and retrieval processes of memory. It should be noted that hippocampal subfields were not assessed in the present study; the findings summarized here are presented as contextual evidence from the broader literature rather than as direct results of this analysis. Despite the fact that the present study measured hippocampal volume as a whole, the difference found for the hippocampus between AD and control subjects is consistent with the hippocampal degeneration found at the subfield level.

#### 4.2.4. Entorhinal Cortex Atrophy as an Early Marker of AD

In Alzheimer’s disease, the entorhinal cortex is affected early and sometimes before the hippocampus. This is supported by the large and similar relative group volume difference in the hippocampus compared to WM and GM. This was able to be identified by using the same normalization and modeling framework.

Neurofibrillary tau pathology is known to start in the earliest stages of the disease in the entorhinal cortex and progress to the hippocampus and then to more extensive cortical regions according to the Braak staging pathology [40,41]. In terms of its function, the entorhinal cortex serves as a crucial gateway between the neocortex and the hippocampal memory circuit to the extent that it is heavily involved in the transport of sensory and related information to the hippocampal memory circuit. Hence, early tau pathology and neuronal loss, leading to entorhinal–hippocampal disruption, are believed to be one of the main causes of early memory impairments in AD [41,42]. In our results, the entorhinal cortex showed a slightly larger percentage difference in AD and CN participants compared to the hippocampus, which supports the idea that they are an early and highly vulnerable site in the disease pathway. MRI studies have also shown that atrophy of the entorhinal cortex may be used to predict conversion from MCI to AD, thus providing clinical relevance to these structural differences [41,43].

In line with this susceptibility, our exponential models estimated the age-associated sectional difference in the entorhinal cortex to be −1.00%/year for the AD group, versus −0.22%/year for the CN group—a between-person model estimate rather than a measured within-subject atrophy rate which was larger than the corresponding age-associated sectional difference in the hippocampus for the AD group compared to the CN group, supporting the proposed role of the entorhinal cortex as an early and highly vulnerable site along the AD pathway [40,41].

#### 4.2.5. Global Gray Matter and White Matter Changes

Under our framework, the present analysis demonstrated that the magnitude of the AD vs. CN difference was greater for GM and cerebral WM, although the separation between the diagnostic groups was not as great as we saw for the medial temporal regions. This trend is consistent with the trend observed by Saha et al. [22]. The lower GM volume was widespread in both groups (AD and mixed dementia) compared to the controls and was most abundant in the inferior temporal gyrus, parahippocampal gyrus, and thalamus. Similarly, the WM differences were fairly widespread, although they were not as regionally specific as GM changes. This is also supported by Salokhiddinov et al., who found that GM volume in healthy subjects was about 18% larger than in mild AD patients using high-resolution 3T MRI scans; the reduction in GM was greater and more widespread than the reduction in WM [44].

Sun et al. (2025) subsequently showed that the decrease in GM volume in patients with AD was strongly correlated with their performance on cognitive and memory scales [45], which further underscored the functional implications of the patterns of GM seen in our dataset. Here, there was a greater GM separation between AD and CN than between WM, which is consistent with the notion that amyloid and tau pathology primarily target GM and that this results in selective neuronal loss. WM changes in AD, on the other hand, may be due to decay in the downstream sections rather than to primary AD pathology, and/or downstream vascular influences may be present. A recent study by Liou et al. (2025) showed that the white matter tracts of healthy controls who were amyloid-positive exhibited myelin degradation, neuroinflammation, and axonal injury, which are all secondary to the cortical pathology of AD and not primary targets of its pathological mechanisms [46]. In support of this interpretation, Tian et al. (2023) demonstrated that WM degeneration pathways in AD are linked to the deposition pattern of tau but not the primary site of amyloid deposition, implying that WM changes may be a component of traveling tau-related injury but not an independent process of the disease [47]. In the present study, the ratio analyses confirm this interpretation, as they reveal that the atrophy observed in the AD group is more specific and not just a consequence of a general reduction in brain volume. This suggests that there is an altered proportional tissue organization and not simply equivalent global GM and WM compartments among people with AD vs. CN. In our exponential models, the AD-associated cross-sectional age difference was also moderately larger for cerebral WM than for CN, while total GM showed comparable decline between groups, consistent with the more diffuse pattern of global tissue change relative to the more regional pattern of medial temporal atrophy described above [22,44].

#### 4.2.6. Brain Volume Fraction and Region-Specific Atrophy

The lower BPF in all AD subjects in the age range indicates a decrease in total brain tissue relative to the intracranial space. Furthermore, AD patients had a decreased hippocampal fraction among total GM. This suggests that the volume loss in the hippocampus was disproportionate even to the overall volume loss of gray matter. Specifically, Xiao et al. (2023) studied the hippocampal parenchymal fraction (HPF) in CN, MCI, and AD participants and found a progressive decrease in HPF from CN to MCI and from MCI to AD [48]. Also, HPF showed strong coupling with cortical gray matter atrophy across the disease continuum. This is directly consistent with what we found: our volume loss was not just attributable to overall GM loss but was a disproportionate loss of GM in the hippocampus.

Another report found that white matter hyperintensities were associated with decreased regional GM volumes in AD, indicating that the structural context of medial temporal structures is also affected by the primary loss of GM volume [49]. Together, these results are in line with known evidence of the hippocampus being severely and selectively affected by the tau and amyloid pathology that is observed in AD.

### 4.3. Sex-Related Findings

From the selected cohort, males and females had age-related decreases in normalized brain volumes. Concurrently, the volumes of the AD group were lower than those of the CN group, for both men and women. Sex-related differences were statistically significant in some areas but were generally smaller and less consistent in direction than differences by age and diagnosis.

The general neuroimaging literature on sex differences in AD and brain aging is also inconclusive. The rate of longitudinal hippocampal differences and cognitive decline has been suggested to be higher in women than in men in AD, regardless of age correction [50]. Previous studies revealed greater overall brain atrophy and poorer performance on neuropsychological measures in women compared to men, which correlated with neuroimaging measures of brain pathology, such as the volume of the hippocampus [51]. Other studies suggest that apparent sex differences in brain volume are much smaller or variable after taking into account intracranial volume, age, and diagnostic status [52].

A relevant example is the analysis in Woodward et al.’s (2024) [27] systematic review. They found that gray matter decline in male and female AD and MCI groups was different over time for certain areas, such as the entorhinal gyrus, thalamus, and temporal areas [27]. They similarly found that both men and women had overall hippocampal atrophy when compared to the healthy controls. One large-scale study of 12,638 longitudinal brain MRIs from 4726 participants across 14 cohorts confirmed that males had greater rates of decline in more regions of the cortex and subcortical structures, but the differences between the sexes were too small to account for the observed sex differences in AD prevalence alone [53]. In other words, this suggests that sex effects can be present but small and non-independent of other factors in structural MRI. Several biological mechanisms have been proposed to explain sex differences in AD risk and progression, including estrogen loss at menopause [54,55,56], age-related testosterone decline [57,58,59], and possible interactions with the APOE4 genotype [60]. None of these variables—hormonal status, menopausal staging, or genotype—were measured in the present study, and we did not observe robust or consistent sex effects in our morphometric data. These mechanisms, therefore, remain possible explanations from the literature rather than interpretations supported by our findings.

### 4.4. BMI Findings and Biomarker Findings

In the current study, BMI and BMI × Diagnosis were mostly not significant. The volume of the hippocampus, entorhinal cortex, and gray matter did not significantly differ by BMI or by the interaction between BMI and diagnosis. The results indicated that BMI was not a significant factor in the differences in brain volume in the group of participants examined.

The results of previous investigations of BMI and brain structure in elderly people have been inconsistent. Increased dementia risk and decreased brain volume have been linked to higher BMI in midlife, but these relationships are less robust or not observed in late life [61]. Moreover, no significant correlation between BMI and several brain neuroimaging measures of Alzheimer’s disease, such as whole-brain volume and white matter hyperintensities (WMH) burden, was found by Lane et al. (2021) [62]. The connection between BMI and brain structure might be impacted by variables like age, metabolic status, vascular health, and disease stage.

In contrast to BMI, plasma pT217 clearly showed a connection to brain changes associated with Alzheimer’s disease. The present study confirmed that plasma pT217 is elevated in AD patients, and plasma pT217 was correlated with decreased volumes of the hippocampus, entorhinal cortex, and total GM. The strongest correlation was found in the hippocampus. This is in line with emerging data indicating pT217 as one of the most sensitive blood-based markers of Alzheimer’s disease. A previous study by Karlsson et al. (2025) revealed that plasma pT217 levels well capture the underlying tau pathology and are strongly correlated with the accumulation of amyloid and tau in the brain [63].

The present study found a negative correlation between pT217 and medial temporal areas, which is of importance as hippocampal volume loss has been a well-established marker of progression of Alzheimer’s disease and cognitive decline. A similar result showing a decrease in hippocampal volume and increased rates of neurodegeneration has also been reported in other studies, in which patients with abnormal levels of pT217/AB42 predicted quicker cognitive decline and hippocampal atrophy [8]. These observations are consistent with the view that increased pT217 levels signal the AD process and that the medial temporal lobe atrophy found in our MRI study is biologically consistent. In summary, the relationship between plasma pT217 and MRI measures of structure highlights the potential for complementary information. pT217 is a measure of underlying tau pathology, whereas the MRI measures the structural consequences; both measures are useful for characterizing AD-related neurodegeneration.

### 4.5. Limitations and Future Directions

This study was subject to several limitations: the number of participants for the filtered cohort’s age range was not evenly distributed, with less data at the extreme ends of the age spectrum. In addition, the number of AD participants was much lower than that of CN, and AD severity was not available. Survivorship bias may also play a role in the data available for older participants aged 80 years and older. Diagnostic status was also assigned at study entry, which was consistent across all participants. Follow-up scans were analyzed under this same classification without being able to confirm later conversion (CN to AD) or stability of AD severity. This introduces the possibility of diagnostic misclassification. Even though repeated scans were included, the work better supports group-level association comparison rather than disease course progression, as that is not solely influenced by MRI volume alone.

Another limitation is that the volume and biomarker matching was more limited compared to the entire MRI cohort. The pT217 subsample (*n* = 289) was considerably smaller than the imaging cohort, and its characteristics relative to the full cohort were not directly compared, raising the possibility of selection bias. Reported BMI and pT217 correlations were also not corrected for multiple comparisons and residual diagnosis, and R^2^ are not reported. A formal power analysis was not conducted for the BMI diagnosis interaction term; given the modest subsample size in some strata, the nonsignificant result should be interpreted with caution regarding statistical power to detect a true effect.

The models mainly focused on group-level associations between diagnosis and ICV-normalized volumes and did not account for genetic status or cognitive performance metrics. Although the use of MRI and centralized UCSFFSX7 data provided a common processing framework, differences in residual heterogeneity and other confounding factors such as other demographics, genetics, other biomarkers, and other clinical factors would nonetheless remain.

Volume loss is not the only clinical marker of AD progression. Evidence-based screening utilizing MRI is a valuable tool; biologic markers and cognitive function testing should be used in conjunction to obtain better insight into disease monitoring and progression. Other factors, such as education, socio-economic status, medical history, and disease onset, play a role in AD severity and progression. These factors may help explain much of the variability seen across the AD group compared to CN.

Work in the future could create more balanced groups, with a larger AD cohort that better represents the full age spectrum evenly. Additionally, incorporating MRI volumes and their association with fluid biomarkers and cognitive function tests may create stronger models to account for individual disease progression.

## 5. Conclusions

This study used 3T MRI data to look at how age, diagnosis, sex, BMI, and plasma tau217 were associated with ICV-normalized brain volumes from a large cohort of participants and scans. Older age and AD diagnosis were consistently associated with lower overall brain volumes for the hippocampus, entorhinal cortex, total GM, and cerebral WM. The entorhinal cortex showed the greatest degree of separation, where AD females and males alike showed approximately a 25% lower value than CN participants. Hippocampus/ICV separation followed and was approximately 21% and 20% lower in AD females and males, respectively. In terms of global measures, total GM had a greater overall lower normalized volume, where AD females were approximately 11% lower, with a similar comparison of 10% lower in AD males. Cerebral WM had the lowest percent mean ROI/ICV difference observed, where females and males were 4% and 5% lower in AD, respectively.

Morphometric ratio-based and relationship-based analyses also supported differences in brain structure among groups. There was a positive association between GM and WM, and the diagnosis interaction suggested that there was a clear statistical difference between AD and CN participants. Also, ICV-normalized hippocampal volume was positively associated with the entorhinal cortex, with a similarly significant diagnosis interaction. Therefore, the data suggest that the medial temporal morphometric relationships have a different scale between AD and CN groups. When the diagnosis x sex interaction was tested, there was no significant difference among the observed ROIs. This points toward the idea that AD-associated volume reduction was broadly comparable among males and females. As expected, plasma tau217 was generally higher in the AD group, and there was a negative trend associated with this biomarker and the morphometric variables. The strongest association was with the hippocampus, followed by the GM and the entorhinal cortex. Cerebral WM had the weakest association. By examining these diagnosis-dependent relationships between the linked areas, along with biological markers, this work extends beyond regional comparisons and provides an integrated characterization of AD morphometric changes.

In all, this work can support the efforts for MRI morphometry by providing valuable insight for characterizing and tracking AD alongside biomarkers and other clinically meaningful parameters. Even though MRI anatomy should not be solely used for diagnostic purposes, ICV-normalized measures can provide insight into how AD is associated with brain volume differences and become valuable markers clinicians can use in diagnosis of the disease. In essence, this work may be useful for characterizing neurodegenerative disease, monitoring morphological impact, and supporting future work that integrates a multitude of disease-related clinical assessments.

## Figures and Tables

**Table 1:** ROI sample sizes for number of scans and participants by sex.

		Males		Females		Total	
		Scans (*n*)	Participants (*n*)	Scans (*n*)	Participants (*n*)	Scans (*n*)	Participants (*n*)
Region of Interest							
	Hippocampus	1578	554	1941	798	3519	1352
	Entorhinal Cortex	1578	554	1941	798	3519	1352
	Total GM	1578	554	1943	800	3521	1354
	Cerebral WM	1574	553	1934	796	3508	1349

**Table 2:** Results output of linear mixed-effects model for age, diagnosis, and sex [ROI/ICV ~ Age_centered_ + Diagnosis + Sex + (1|PTID)].

**Hippocampus/ICV**				
**Predictor**		**β Estimate**	**95% CI**	***p* Value**
	Age	−4.22 × 10^−5^	−4.54 × 10^−5^ to −3.9 ×10^−5^	<0.001
	AD vs. CN	−1.02 × 10^−3^	−1.11 ×10^−3^ to −9.54 × 10^−4^	<0.001
	Male vs. Female	−2.03 × 10^−4^	−2.67 × 10^−4^ to −1.38 × 10^−4^	<0.001
**Entorhinal Cortex/ICV**				
**Predictor**		**β Estimate**	**95% CI**	***p* Value**
	Age	−1.96 × 10^−5^	−2.20 × 10^−5^ to −1.72 × 10^−5^	<0.001
	AD vs. CN	−5.81 × 10^−4^	−6.38 × 10^−4^ to −5.25 × 10^−4^	<0.001
	Male vs. Female	8.26 × 10^−5^	3.4 × 10^−5^ to 1.31 × 10^−4^	<0.001
**Total GM/ICV**				
**Predictor**		**β Estimate**	**95% CI**	***p* Value**
	Age	−1.38 × 10^−3^	−1.58 × 10^−3^ to −1.19 × 10^−3^	<0.001
	AD vs. CN	−0.041	−0.045 to −0.037	<0.001
	Male vs. Female	−9.34 × 10^−3^	−0.013 to −5.67 × 10^−3^	<0.001
**Cerebral WM/ICV**				
**Predictor**		**β Estimate**	**95% CI**	***p* Value**
	Age	−1.92 × 10^−3^	−2.09 × 10^−3^ to −1.76 × 10^−3^	<0.001
	AD vs. CN	−0.013	−0.016 to −9.01 × 10^−3^	<0.001
	Male vs. Female	5.56 × 10^−3^	2.39 × 10^−3^ to 8.71 × 10^−3^	<0.001

**Table 3:** Exponential model equations for age-ROI/ICV-associated trends in CN and AD groups.

ROI		CN Equation	AD Equation
	Hippocampus	y = 0.0089583e^(−0.0073Age)^	y = 0.0072432e^(−0.0077Age)^
	Entorhinal	y = 0.0029401e^(−0.0022Age)^	y = 0.0039587e^(−0.0101Age)^
	Total GM	y = 0.54824e^(−0.0040Age)^	y = 0.48192e^(−0.0038Age)^
	Cerebral WM	y = 0.40605e^(−0.0045Age)^	y = 0.44011e^(−0.0063Age)^

**Table 4:** Estimated age-associated annual percent change for ROI/ICV from exponential models in CN and AD groups.

ROI		CN Decline (% Per Year)	AD Decline (% Per Year)
	Hippocampus	−0.73	−0.77
	Entorhinal	−0.22	−1.00
	Total GM	−0.40	−0.38
	Cerebral WM	−0.45	−0.62

**Table 5:** Mixed-effects model evaluating the association of ICV-normalized volume relationships. PTID was used as a random intercept to incorporate multiple MRI scans for a single participant. CN is the reference group for diagnosis.

Model		Predictor	β Estimate	95% CI	*p* Value
	GM as function of WM	Cerebral WM	0.783	0.748 to 0.818	<0.001
	GM function as WM	Cerebral WM × diagnosis	0.338	0.287 to 0.389	<0.001
	Hippocampus as function of entorhinal cortex	Entorhinal cortex	0.601	0.558 to 0.643	<0.001
	Hippocampus as function of entorhinal cortex	Entorhinal cortex × diagnosis	0.447	0.366 to 0.529	<0.001

**Table 6:** Spearman correlations for plasma pT217 and normalized MRI volumes.

ROI		*N*	Spearman	*p* Value
	Hippocampus	289	−0.402	<0.001
	Entorhinal Cortex	289	−0.303	<0.001
	Total GM	289	−0.341	<0.001
	Cerebral WM	289	−0.116	0.049

## Data Availability

Restrictions apply for the availability of these data. Data were obtained from ADNI and are available through the ADNI Image and Data Archive (https://adni.loni.usc.edu/) upon approval. In accordance with the ADNI Data Use Agreement, individual participant-level data cannot be redistributed by the authors.

## Abbreviations

AD	Alzheimer’s disease
ADNI	Alzheimer’s Disease Neuroimaging Initiative
Aβ	Amyloid-beta
APOE4	Apolipoprotein E ε4 allele
ARC	Analysis Ready Cohort
BMI	Body mass index
BPF	Brain parenchymal fraction
CA1	Cornu ammonis 1
CN	Cognitively normal
CI	Confidence interval
DOB	Date of birth
GM	Gray matter
HPF	Hippocampal parenchymal fraction
ICV	Intracranial volume
MCI	Mild cognitive impairment
MMSE	Mini-Mental State Examination
MRI	Magnetic resonance imaging
pT217	Plasma phosphorylated tau 217
PTGENDER	ADNI variable for participant sex
PTID	ADNI variable for participant identification number
ROI	Region of interest
UCSF	University of California, San Francisco
VBM	Voxel-based morphometry
WM	White matter
